# Optimally mapping multivariate regional brain age and clinical severity patterns in Alzheimer's disease using AgeNet‐SHAP explainable AI approach

**DOI:** 10.1002/alz70856_101234

**Published:** 2025-12-25

**Authors:** Gauri Darekar, Taslim Murad, Hui‐Yuan Miao, Deepa S. Thakuri, Ganesh Chand

**Affiliations:** ^1^ Washington University in St. Louis, St. Louis, MO, USA; ^2^ University of Missouri, Columbia, MO, USA

## Abstract

**Background:**

Age is a significant risk factor for mild cognitive impairment and Alzheimer's disease (MCI/AD), and identifying brain age patterns is critical for comprehending the normal aging and MCI/AD processes. Prior studies have widely established the univariate relationships between brain regions and age, while multivariate associations remain largely unexplored.

**Method:**

In this study, we utilized many artificial intelligence (AI) methods to estimate brain age using the regional brain volumetric changes gained from MRI. Then the optimal AI model was integrated with the Shapley additive explanations (SHAP) technique to identify the significant multivariate brain regions involved in this prediction. Our methodology was validated using semi‐simulated data (*n* = 187) prior to applying on experimental data (*n* = 668; age range 55.1‐91.5 years; 46.1% females). Moreover, the clinical severity of MCI/AD, measured with Clinical Dementia Rating‐Sum of Boxes (CDR‐SB), was investigated within the framework of optimal model combined with SHAP.

**Result:**

Our results indicated that the deep learning model (AgeNet) tremendously outperformed the conventional ML models for brain age prediction, and AgeNet integrated with SHAP (AgeNet‐SHAP) identified all ground‐truth perturbed regions as key predictors of brain age in simulation. In the experimental dataset, compared to CN, MCI exhibited moderate differences in brain regions, whereas AD had highly robust and widely distributed regional differences. The individualized AgeNet‐SHAP regional features further showed the associations with clinical severity scores in the AD continuum, thereby highlighting disease‐specific and normal aging‐specific differential regions.

**Conclusion:**

The explainable AI method, AgeNet‐SHAP, demonstrated good performance in brain age prediction based on a large MRI dataset, along with identifying the hierarchy of multivariate brain regional associations with this prediction. Additionally, this method captured the brain regional associations with AD severity. These results collectively facilitate data‐driven predictive modelling approaches for disease progression, diagnostics, prognostics, and personalized medicine efforts.

